# Modified Liuwei Dihuang Decoction Ameliorates Oligoasthenozoospermia in Mice via Modulation of the PI3K/AKT/Nrf2 Signaling Pathway

**DOI:** 10.3390/ph18091363

**Published:** 2025-09-12

**Authors:** Mingzhu Li, Linhuang Chen, Haotian Xu, Junlin Li, Yatian Liu, Xiuyun Chen, Minyi Luo, Xinyuan Xie, Mingyu Yin, Jinyang He

**Affiliations:** 1 Science and Technology Innovation Center, Guangzhou University of Chinese Medicine, Guangzhou 510405, China; mingzhu2025@foxmail.com (M.L.); 15973335192@163.com (L.C.); xhaotian0529@163.com (H.X.); lijunlin555@foxmail.com (J.L.); yatianliu9696@163.com (Y.L.); 15207688546@163.com (X.C.); 13760650651@163.com (M.L.); ymy6528127@163.com (M.Y.); 2 Artemisinin Research Center, Guangzhou University of Chinese Medicine, Guangzhou 510405, China; xin2472707780@163.com

**Keywords:** Modified Liuwei Dihuang Decoction, Oligoasthenozoospermia, oxidative stress, inflammation, PI3K/AKT/Nrf2 signaling pathway

## Abstract

**Background**: Oligoasthenozoospermia (OA) is a common cause of male infertility. Modified Liuwei Dihuang Decoction (MLWDH) is an improved version of Liuwei Dihuang Decoction (LWDH), a traditional Chinese medicine prescription, which has demonstrated significant therapeutic effects against OA. This study aims to evaluate the protective effects of MLWDH against OA and elucidate its underlying molecular mechanisms. **Methods**: The constituents of MLWDH were identified via UPLC-HRMS and compound databases (TCMSP, HERB). Network pharmacology analysis was conducted to predict potential therapeutic targets and associated signaling pathways. In vivo, a CP-induced mouse model of OA was established to evaluate the therapeutic efficacy of MWDH by assessing testicular and epididymal indices, sperm quality, histopathological changes and serum hormone levels. Oxidative stress markers, including MDA, SOD, GSH and NO, were measured using commercial assay kits. The underlying molecular mechanisms, particularly those related to oxidative stress and inflammation (PI3K, Akt, Nrf2, Keap1, HO-1, NQO1, NF-κB, TNF-α, IL-6), were further elucidated by RT-qPCR, Western blot, and immunofluorescence. **Results**: A total of 345 major bioactive compounds were identified in MLWDH. Network pharmacology and molecular docking analyses indicated that MLWDH exerts its effects primarily through the PI3K/AKT signaling pathway. MLWDH administration in vivo significantly improved sperm count, motility, and morphology, while also increasing serum levels of testosterone, FSH, and LH. Moreover, MLWDH significantly mitigated oxidative damage, as evidenced by decreased MDA concentrations and elevated levels of GSH, NO and SOD. Mechanistic investigations further substantiated that MLWDH enhanced PI3K/AKT/Nrf2 signaling while inhibiting NF-κB signaling in OA mice. **Conclusions**: Our findings suggest that MLWDH ameliorates OA in a preclinical mouse model by improving sperm quality and testicular function, potentially via activation of the PI3K/AKT/Nrf2 signaling pathway and the inhibition of NF-κB signaling, thereby alleviating oxidative stress and inflammatory responses.

## 1. Introduction

Male factor infertility accounts for approximately 50% of reproductive failures [1]. Impairments in sperm quality are major contributors to male infertility, with oligospermia and asthenospermia, defined as reductions in sperm count and motility, respectively, being among the most frequently observed conditions. Epidemiological studies have shown that these abnormalities are involved in approximately 21–51% of cases [2]. This combined manifestation is clinically referred to as Oligoasthenozoospermia (OA), a prevalent and challenging subtype of male infertility that requires effective therapeutic strategies [3].

OA arises from multifactorial etiologies, including oxidative stress, genetic predisposition, environmental exposures, infections, and drug-induced toxicity [4]. Mounting evidence establishes oxidative stress as a critical pathological factor contributing to male infertility, with particular relevance to OA [5]. Functioning as the central orchestrator of cellular redox homeostasis, nuclear factor erythroid 2-related factor 2 (Nrf2) activates a transcriptional program that strengthens the antioxidant capacity of cells. Previous studies have demonstrated that Nrf2 deficiency results in impaired spermatogenesis, testicular atrophy and decreased sperm quality, demonstrating its essential role in maintaining reproductive function [6,7]. Under oxidative stress, Nrf2 becomes activated through its dissociation from Kelch-like ECH-associated protein 1 (Keap1), allowing its translocation into the nucleus [8]. Following nuclear translocation, Nrf2 interacts with antioxidant response elements (ARE) to upregulate a battery of antioxidant and cytoprotective genes [8,9,10]. The phosphoinositide 3-kinase/protein kinase B (PI3K/AKT) pathway is a crucial upstream regulator of Nrf2 activation. Activation of PI3K/AKT not only facilitates Nrf2 nuclear translocation but also supports cell survival under oxidative stress [11]. In the testis, this pathway contributes to the proliferation of spermatogenic cells, enhances Sertoli cell viability, and maintains the integrity of the blood-testis barrier, thereby ensuring the proper progression of spermatogenesis [12]. The coordinated interplay between PI3K/AKT and Nrf2 signaling represents promising therapeutic targets for alleviating OA [9].

Current clinical guidelines for male infertility offer limited pharmacological options targeting spermatogenic dysfunction. Although assisted reproductive technologies are effective in selected cases, they are constrained by high costs and modest overall success rates [13], highlighting the urgent need for alternative therapeutic approaches. TCM has gained attention as a complementary strategy for treating OA due to its multi-targeted actions, clinical efficacy, and favorable safety profile [14]. Recent evidence further substantiates the therapeutic potential of TCM in OA. A meta-analysis demonstrated that the classical formula Wuzi Yanzong significantly improves semen quality parameters and increases partner pregnancy rates in men with abnormal sperm parameters [15]. Mechanistic investigations further demonstrate that TCM formulations can modulate key redox and survival pathways implicated in OA, including PI3K-AKT signaling and stress-activated MAPKs [3]. Moreover, systematic syntheses indicate that combining TCM with conventional agents such as L-carnitine may confer additive benefits on semen parameters, highlighting TCM’s potential to complement established therapies and broaden treatment options for OA [16]. LWDH was originally recorded in *Xiaoer Yaozheng Zhijue* by Qian Yi during the Song Dynasty [17]. It is widely recognized for its therapeutic efficacy in nourishing kidney Yin and has been extensively applied in the treatment of various disorders related to kidney deficiency, including diabetes [18], nephropathy [19], polycystic ovary syndrome [20], and infertility [21]. A recent clinical study has demonstrated that LWDH can improve sperm viability and treat male infertility [22], while a preclinical study has shown that LWDH alleviates testicular inflammation in aging rat models [23]. However, comprehensive investigations into its therapeutic efficacy for OA remain limited. Although LWDH is widely applied in clinical settings, it does not sufficiently address the complex pathophysiology of OA, which involves both kidney deficiency and qi stagnation [24,25], according to the Traditional Chinese Medicine (TCM) theory. Therefore, its efficacy may be limited. To improve its efficacy, a modified version, MLWDH, has been developed by incorporating additional herbal components. This modification was intended to reinforce the therapeutic efficacy of LWDH by targeting both deficiency and stagnation syndromes. Additional herbs such as *Bupleurum chinense DC* (Chaihu) [26], *Citrus medica* L. *var. sarcodactylis* (*Noot.*) *Swingle* (Foshou) [27] and *Scolopendra subspinipes mutilans* L. *Koch* (Wugong) [28] are incorporated to soothe liver qi, promote blood circulation and dispel stasis. The chemical constituents of *Cinnamomum cassia* (L.) *J. Presl* (Rougui), *Lycium barbarum* L. (Gouqi), and *Angelica sinensis (Oliv.) Diels* (Danggui) have been reported to enhance erectile function [29], safeguard sperm structural integrity [30], and promote testosterone secretion by Leydig cells [31]. Notably, our preliminary experiments revealed that MLWDH demonstrated significantly greater therapeutic efficacy than the classical LWDH in improving key parameters related to OA. Preliminary clinical applications have indicated that MLWDH may enhance male reproductive capacity and increase the likelihood of natural conception, highlighting its potential as a promising therapeutic strategy for OA. However, the collaborative mechanism through which the MLWDH operates in alleviating OA remains to be fully elucidated.

We integrated UPLC-HRMS-based non-targeted metabolomics with public databases and literature mining to identify key active constituents of MLWDH, surpassing the limitations of traditional network pharmacology alone. To substantiate the predicted pharmacological relevance of MLWDH, we conducted in vivo studies to evaluate its therapeutic efficacy against reproductive impairs and decipher its molecular mechanisms.

## 2. Result

### 2.1. Bioactive Components and Therapeutic Targets of MLWDH Identified by UPLC-HRMS and Network Pharmacology

#### 2.1.1. Characterization of Principal Constituents in MLWDH via UPLC-HRMS Analysis

**Figure 1:**
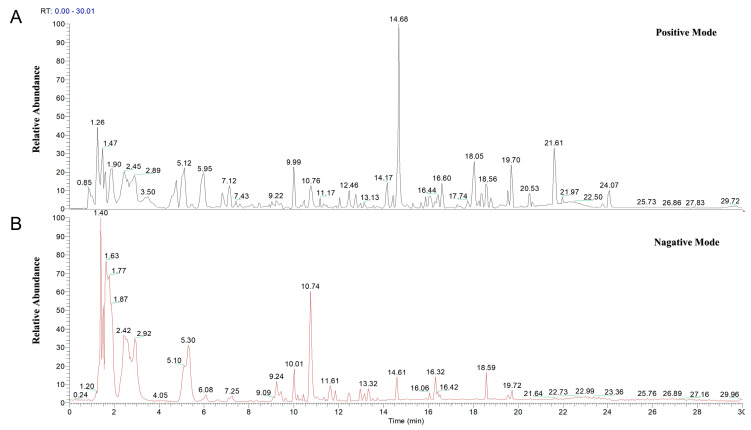
Representative total ion chromatograms (TICs) of MLWDH detected by UPLC-HRMS, acquired in (**A**) positive and (**B**) negative electrospray ionization modes.

**Figure 2:**
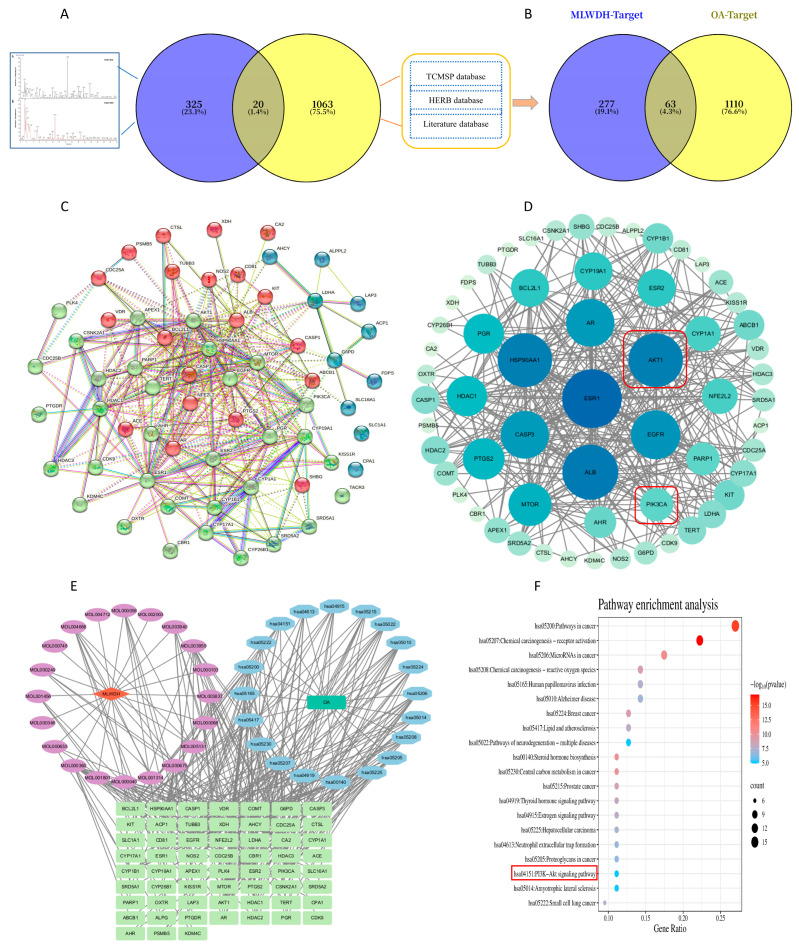
Network pharmacology profiling of MLWDH in OA. Screening of representative compounds from MLWDH based on UPLC-HRMS results and database mining (**A**). Overlapping targets between MLWDH-related compounds and OA-associated genes (**B**), PPI protein interaction network (**C**). Key targets of MLWDH against OA (**D**). Drug-compound-target-disease network diagram (**E**). KEGG pathway enrichment analysis (**F**).

To elucidate the pharmacologically active components of MLWDH, UPLC-HRMS analysis was conducted, laying a chemical foundation for subsequent network pharmacology and mechanistic studies. 345 compounds were identified from the MLWDH extract via UPLC-HRMS profiling (Figure 1), including diverse classes such as organic acids, flavonoids, and phenolic acids. In parallel, 1083 candidate compounds were retrieved from existing literature and public TCM databases. By cross-referencing the UPLC-HRMS results with these database-derived compounds, 20 overlapping constituents were identified (Figure 2A), representing the core active ingredients potentially responsible for the observed pharmacological effects of MLWDH. Among them, methyl cinnamate fully met the ADME-related criteria (oral bioavailability, OB ≥ 30%; drug-likeness, DL ≥ 0.18 in TCMSP), while the other 19 compounds have been consistently reported to exert activities related to oxidative stress modulation or inflammation regulation, which are central to the present study [32,33,34,35,36,37,38,39,40,41,42,43,44,45,46,47,48,49,50]. Detailed chemical information of these representative components is provided in Table 1.

#### 2.1.2. Identification and Prioritization of Putative Targets of MLWDH Against OA

Identifying the intersection between compound-related and disease-associated targets is essential for clarifying the therapeutic mechanisms of MLWDH against OA. The 20 active compounds in MLWDH were subjected to target prediction analysis via the SwissTargetPrediction platform, and after removing redundancies, 340 unique protein targets were obtained. Additionally, 1173 non-redundant OA-associated targets were screened from the GeneCards database. After overlapping the predicted compound targets with disease-related targets, a total of 63 common genes were recognized as therapeutic mediators potentially involved in the pharmacological activity of MLWDH against OA (Figure 2B).

To elucidate the interrelationships between the 63 common genes, a PPI network was generated via the STRING platform and graphically represented using Cytoscape software (version 3.10.3) (Figure 2C). Topological analysis based on degree centrality was performed to rank the nodes, where a higher degree indicated a greater number of interacting partners. In the network, the relative degree values were visually represented by the color intensity of the elliptical nodes, with darker shades indicating higher connectivity. Further analysis using the CytoNCA plugin enabled the extraction of a subnetwork comprising 20 hub genes, including ESR1, HSP90AA1, AKT1, and PIK3CA, which demonstrated the highest centrality scores and may serve as significant mediators of the therapeutic effect of MLWDH (Figure 2D). Notably, AKT1 ranked fourth and PIK3CA ranked eighteenth in terms of degree, highlighting their potential involvement in the regulatory mechanism. These findings are consistent with subsequent experimental validations, supporting their critical roles in the pharmacological activity of MLWDH.

#### 2.1.3. Network Construction of Drug-Compound–Target–Disease Associations

To visualize the compound–target–disease relationships, a comprehensive interaction network was generated (Figure 2E), comprising 101 nodes and 303 edges. This network highlighted the connectivity between 20 key bioactive compounds and their corresponding protein targets associated with OA.

#### 2.1.4. KEGG Pathway Enrichment Analysis of Putative Targets

Pathway enrichment of the intersecting targets was performed using the KEGG database, revealing key signaling cascades potentially involved in MLWDH’s therapeutic effects on OA. The 20 most statistically significant signaling pathways (*p* < 0.05) are presented in Figure 2F. The enrichment results indicated that the PI3K/AKT signaling is potentially involved in orchestrating the therapeutic effects of MLWDH against OA.

#### 2.1.5. Molecular Docking Analysis of MLWDH Bioactive Compounds with PI3K/AKT Signaling

CytoNCA analysis identified five compounds, including ferulic acid, 7-hydroxy-6-methoxy-2H-chromen-2-one, limonin, oleic acid and esculetin, as the most connected nodes, suggesting their potential central roles in the therapeutic effect of MLWDH. Molecular docking analysis revealed that these compounds presented favorable binding affinities with the selected core proteins (PIK3CA and AKT1), with binding energies ranging from −5.8 to −10.4 kcal/mol (Table 2). Specifically, limonin and esculetin demonstrated the strongest binding affinity with both targets (binding energy ≤ −6.9 kcal/mol), indicating a high likelihood of stable interactions and regulatory potential within the PI3K/AKT pathway. Pymol software (version 3.1.5.1) was utilized for visualization (Figure S1).

### 2.2. MLWDH Alleviates CP-Induced Reproductive Organ Injury and Testicular Histopathology

**Figure 3:**
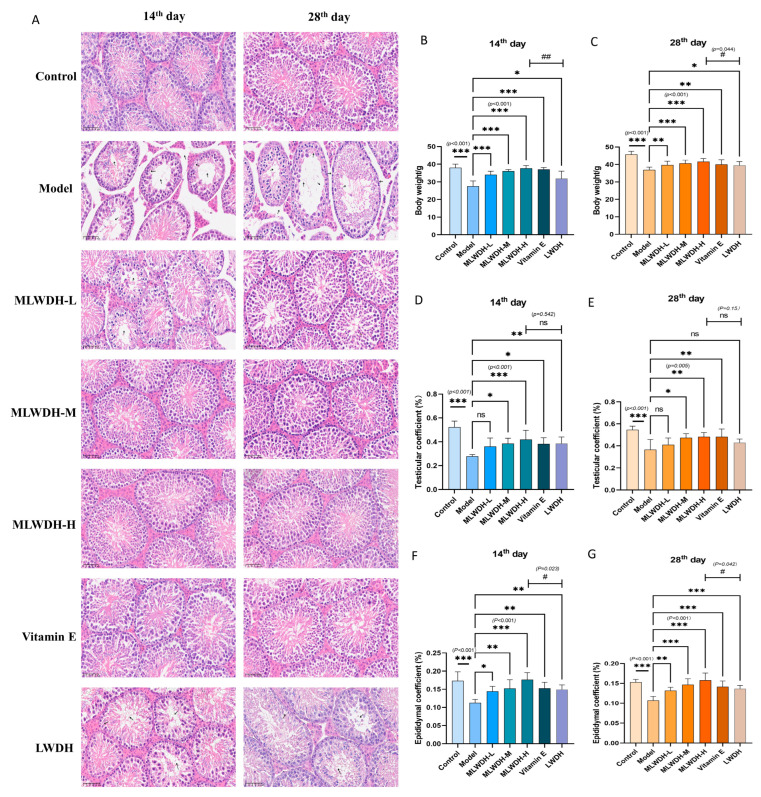
Effects of MLWDH on testicular issues in OA mice. H&E-staining testis of mice (**A**), Scale bar = 50 μm, body weight of mice ((**B**), 14th day; (**C**), 28th day), representative photographs of the testicular morphology (**A**), testicular index ((**D**), 14th day; (**E**), 28th day) and epididymal index ((**F**), 14th day; (**G**), 28th day); 14th day: on the day 14 after modeling;28th day: on the day 28 after modeling. Control: control group, MLWDH-L: low-dose MLWDH-treated group, MLWDH-M: medium-dose MLWDH-treated group, MLWDH-H: high-dose MLWDH-treated group. LWDH: LWDH-treated group; Vitamin E: Vitamin E-treated group. Quantitative data are expressed as mean ± SD (n = 8). Statistical comparisons were performed by one-way ANOVA with the following significance thresholds: ns, no significant, * *p* < 0.05, ** *p* < 0.01, *** *p* < 0.001, versus model group, ^#^ *p* < 0.05, ^##^ *p* < 0.01, versus MLWDH-H group.

To assess the effects of MLWDH in reversing reproductive toxicity, mice were administered low, medium, and high doses of the decoction. As shown in Figure 3B–G, CP exposure resulted in marked reductions in weight (14th day: *p* < 0.001, η^2^ = 0.27; 28th day: *p* < 0.001, η^2^ = 0.43), testicular index (14th day: *p* < 0.001, η^2^ = 0.64; 28th day: *p* < 0.001, c= 0.53), and epididymal index (14th day: *p* < 0.001, η^2^ = 0.58; 28th day: *p* < 0.001, η^2^ = 0.66) compared with the control group. Additionally, MLWDH administration significantly attenuated weight loss and dose-dependently restored both testicular and epididymal indices, suggesting a beneficial effect on reproductive organ mass. To further investigate underlying histopathological changes, H&E staining was performed. CP-induced mice exhibited severe structural damage in the testes, including reduced germ cell numbers, disorganized seminiferous epithelium, interstitial cell depletion, and sloughing of spermatogenic cells. Conversely, MLWDH-treated mice displayed notable preservation of testicular architecture, with increased germ cell density and orderly cellular arrangement (Figure 3A), indicating mitigation of CP-induced testicular degeneration.

### 2.3. MLWDH Increases Reproductive Hormone Levels in OA Mice

**Figure 4:**
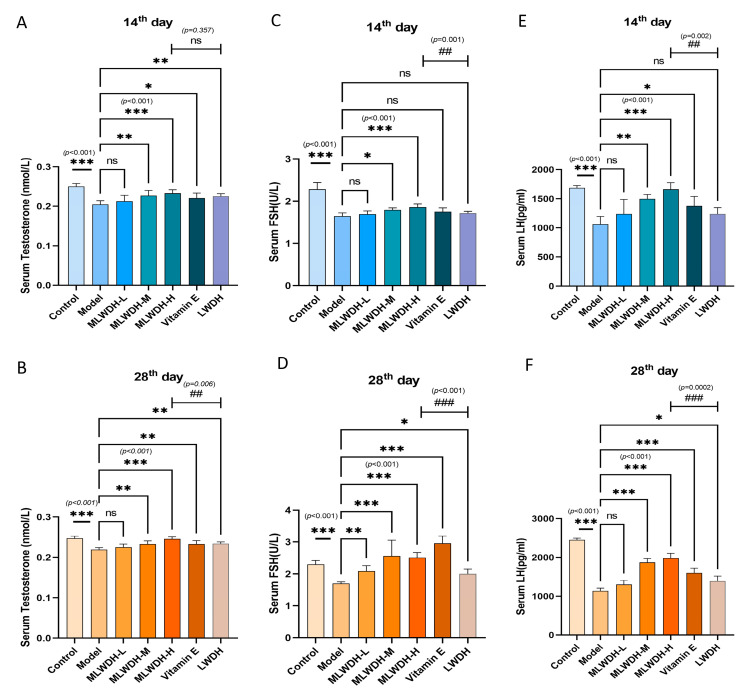
Effects of MLWDH on reproductive hormone levels in the OA mouse model. Testosterone (T; (**A**), 14th day; (**B**), 28th day), follicle-stimulating hormone (FSH; (**C**), 14th day; (**D**), 28th day), and luteinizing hormone (LH; (**E**), 14th day; (**F**), 28th day) levels were measured by ELISA. 14th day: on the day 14 after modeling; 28th day: on the day 28 after modeling. Control: control group, MLWDH-L: low-dose MLWDH-treated group, MLWDH-M: medium-dose MLWDH-treated group, MLWDH-H: high-dose MLWDH-treated group. LWDH: LWDH-treated group; Vitamin E: Vitamin E-treated group. Quantitative data are expressed as mean ± SD (n = 8). Statistical comparisons were performed by one-way ANOVA with the following significance thresholds: ns, no significant, * *p* < 0.05, ** *p* < 0.01, *** *p* < 0.001, versus model group, ^##^ *p* < 0.01, ^###^ *p* < 0.001, versus MLWDH-H group.

Given that disrupted reproductive hormone homeostasis is a major contributor to impaired spermatogenesis, we assessed the concentrations of testosterone, FSH, and LH in serum samples from OA mice. Compared with the control group, CP intervention markedly decreased all three hormones at Day 14 after modeling (T: *p* < 0.001, η^2^ = 0.64; LH: *p* < 0.001, η^2^ = 0.75; FSH: *p* < 0.001, η^2^ = 0.61), a trend that persisted to Day 28 as shown in Figure 4. MLWDH administration reversed these hormonal declines at both Day 14 and Day 28 after modeling. Notably, high-dose MLWDH exhibited superior efficacy in restoring hormonal levels compared to both classical LWDH and Vitamin E, indicating enhanced endocrine-regulating potential.

### 2.4. MLWDH Improves Sperm Quality and Reduces Morphological Abnormalities in OA Mice

**Figure 5:**
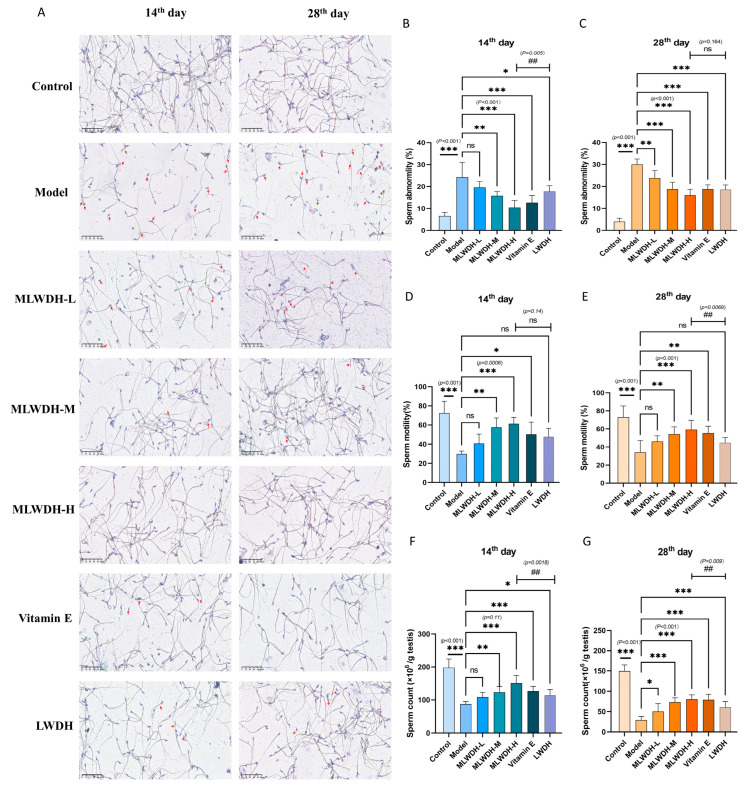
Effects of MLWDH on sperm quality in a CP-Induced OA mouse model. Representative photographs of sperm morphology, Scale bar = 50 μm (**A**), sperm abnormality ((**B**), 14th day; (**C**), 28th day), Sperm motility ((**D**), 14th day; (**E**), 28th day), Sperm count ((**F**), 14th day; (**G**), 28th day). 14th day: on the day 14 after modeling; 28th day: on the day 28 after modeling. Quantitative data are expressed as mean ± SD (n = 8). Control: control group, MLWDH-L: low-dose MLWDH-treated group, MLWDH-M: medium-dose MLWDH-treated group, MLWDH-H: high-dose MLWDH-treated group. LWDH: LWDH-treated group; Vitamin E: Vitamin E-treated group. Statistical comparisons were performed by one-way ANOVA with the following significance thresholds: ns, no significant, * *p* < 0.05, ** *p* < 0.01, *** *p* < 0.001, versus model group, ^##^ *p* < 0.01, versus MLWDH-H group).

To investigate the ameliorative impact of MLWDH against CP-triggered reproductive injury, sperm quality was comprehensively evaluated. CP administration resulted in a significant increase in morphologically abnormal sperm (14th day: *p* < 0.001, η^2^ = 0.75; 28th day: *p* < 0.001, η^2^ = 0.65, Figure 5B,C), particularly exhibiting head folding and midpiece deformities (Figure 5A). This was accompanied by significantly decreased sperm motility (14th day: *p* < 0.001, η^2^ = 0.69; 28th day: *p* < 0.001, η^2^ = 0.63, Figure 5D,E) and total sperm count (14th day: *p* < 0.001, η^2^ = 0.78; 28th day: *p* < 0.001, η^2^ = 0.88, Figure 5F,G) relative to the control group. Notably, MLWDH dose-dependently restored spermatogenic function through coordinated improvement in reduced sperm malformation rates and improved sperm motility and count. The efficacy of high-dose MLWDH was comparable to that of classical LWDH and Vitamin E group.

### 2.5. MLWDH Attenuates Oxidative Stress in OA Mice

**Figure 6:**
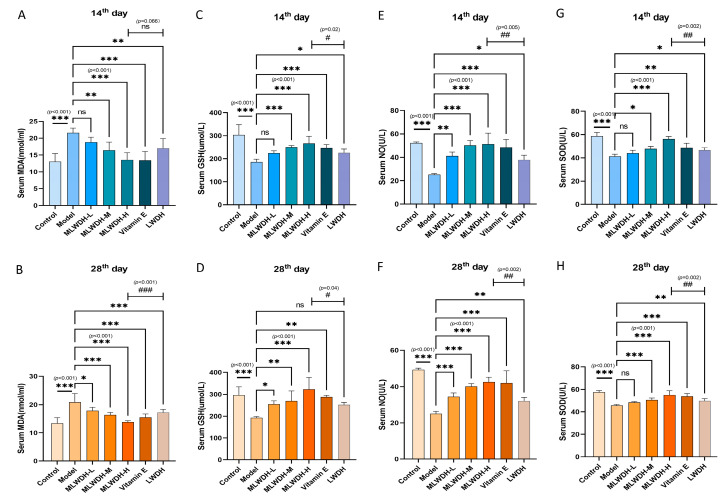
Effects of MLWDH on serum oxidative stress marker levels in OA mice. Levels of malondialdehyde (MDA; (**A**), 14th day; (**B**), 28th day), glutathione (GSH; (**C**), 14th day; (**D**), 28th day), nitric oxide (NO; (**E**)), 14th day; (**F**), 28th day), and superoxide dismutase (SOD; (**G**), 14th day; (**H**), 28th day). 14th day: on the day 14 after modeling; 28th day: on the day 28 after modeling. Control: control group, MLWDH-L: low-dose MLWDH-treated group, MLWDH-M: medium-dose MLWDH-treated group, MLWDH-H: high-dose MLWDH-treated group. LWDH: LWDH-treated group; Vitamin E: Vitamin E-treated group. Quantitative data are expressed as mean ± SD (n = 8). Statistical comparisons were performed by one-way ANOVA with the following significance thresholds: ns, not significant, * *p* < 0.05, ** *p* < 0.01, *** *p* < 0.001, versus model group, ^#^ *p* < 0.05, ^##^ *p* < 0.01, ^###^ *p* < 0.001, versus MLWDH-H group.

Considering the critical influence of oxidative stress in impairing spermatogenesis, we quantified representative biomarkers (MDA, GSH, SOD, and NO) to characterize the therapeutic potential of MLWDH on redox homeostasis. CP administration resulted in a pronounced elevation in serum MDA levels (14th day: *p* < 0.001, η^2^ = 0.67; 28th day: *p* < 0.001, η^2^ = 0.71, Figure 6A,B), indicating elevated lipid peroxidation and systemic oxidative stress. Concurrently, CP significantly suppressed the activities of key antioxidant markers, including GSH (14th day: *p* < 0.001, η^2^ = 0.73; 28th day: *p* < 0.001, η^2^ = 0.63), NO (14th day: *p* < 0.001, η^2^ = 0.69; 28th day: *p* < 0.001, η^2^ = 0.58) and SOD ((14th day: *p* < 0.001, η^2^ = 0.66; 28th day: *p* < 0.001, η^2^ = 0.78, Figure 6C–H). Treatment with MLWDH effectively reversed these alterations, evidenced by reduced MDA levels and restoration of GSH, NO, and SOD activities. These antioxidative effects remained consistent between day 14 and day 28 post-modeling. Notably, the high-dose MLWDH confers superior antioxidant protection compared to both LWDH and the Vitamin E group, highlighting its enhanced efficacy in mitigating CP-induced oxidative damage, likely via more effective reinforcement of endogenous redox defense systems.

### 2.6. MLWDH Activates the PI3K/AKT Signaling Pathway in OA Mice

**Figure 7:**
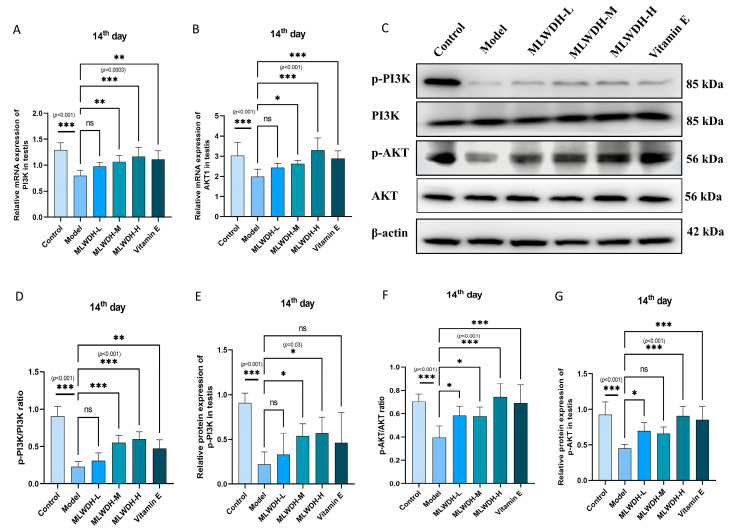

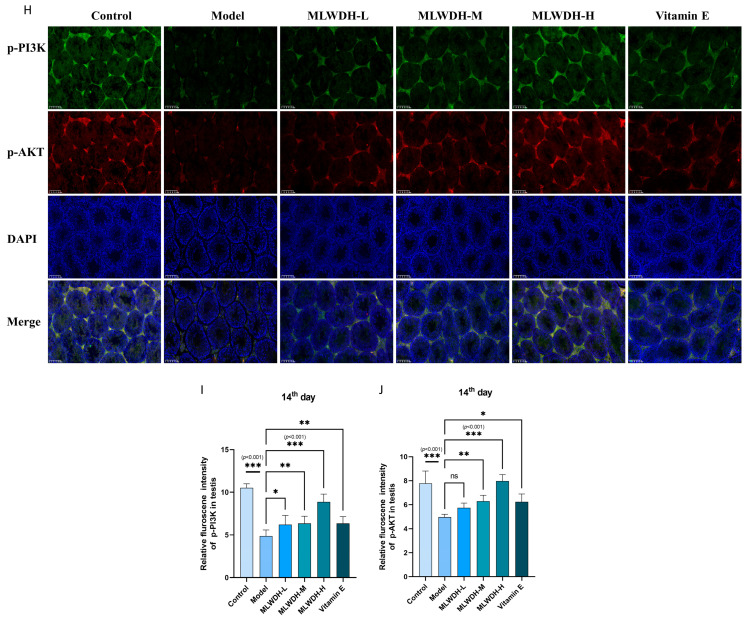
Effects of MLWDH on PI3K/AKT signaling in OA mice on day 14 after modeling. PI3K and Akt1 mRNA expression in testis (**A**,**B**). Representative immunoblots of p-PI3K, PI3K, p-Akt and Akt (**C**). Quantification of p-PI3K (phosphorylated/total) (**D**,**E**) and p-Akt (phosphorylated/total) (**F**,**G**) expression, normalized to GAPDH. Immunofluorescent staining of p-PI3K(green) and p-Akt (red) in testis sections (**H**), Scale bar = 100 μm. DAPI: blue, target proteins: green/red, scale bars: white. Quantification of p-PI3K (**I**) and p-Akt (**J**) protein expression in immunofluorescence. Control: control group, MLWDH-L: low-dose MLWDH-treated group, MLWDH-M: medium-dose MLWDH-treated group, MLWDH-H: high-dose MLWDH-treated group; Vitamin E: Vitamin E-treated group. Quantitative data are expressed as mean ± SD (n = 8). Statistical comparisons were performed by one-way ANOVA with the following significance thresholds: ns, no significant, * *p* < 0.05, ** *p* < 0.01, *** *p* < 0.001, versus model group.

**Figure 8:**
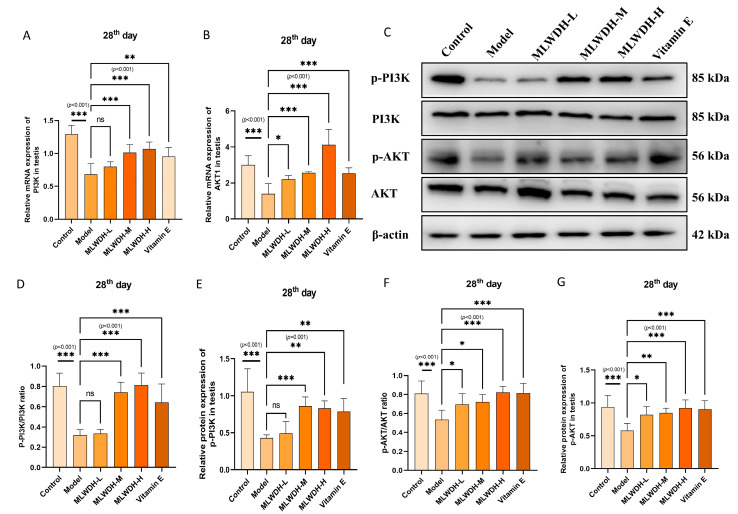

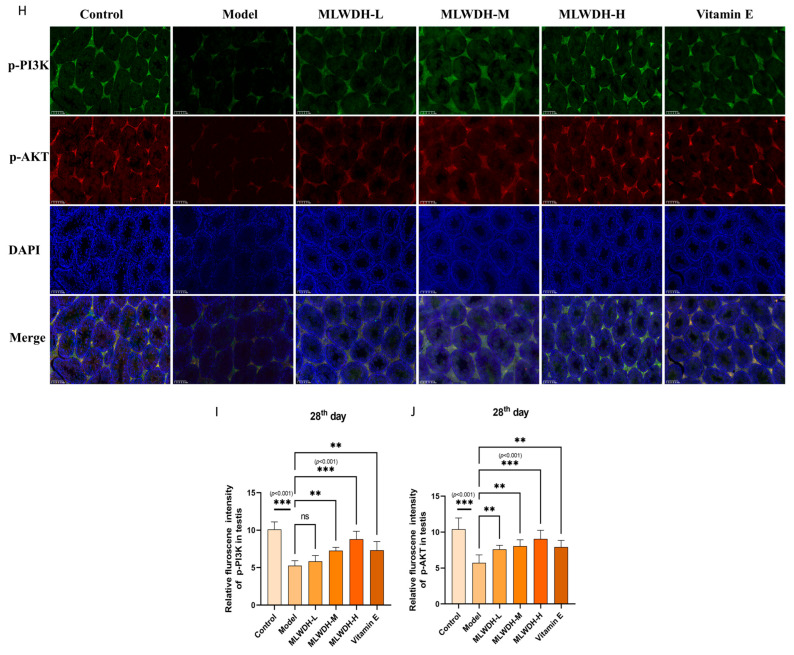
Effects of MLWDH on PI3K/AKT signaling in OA mice on day 28 after modeling. PI3K and Akt1 mRNA expression in testis (**A**,**B**). Representative immunoblots of p-PI3K, PI3K, p-Akt, and Akt (**C**). Quantification of p-PI3K (phosphorylated/total) (**D**,**E**) and p-Akt (phosphorylated/total) (**F**,**G**) expression, normalized to GAPDH. Immunofluorescent staining of p-PI3K (green) and p-Akt (red) in testis sections (**H**), Scale bar = 100 μm. DAPI: blue, target proteins: green/red, scale bars: white. Quantification of p-PI3K (**I**) and p-Akt (**J**) protein expression in immunofluorescence. Control: control group, MLWDH-L: low-dose MLWDH-treated group, MLWDH-M: medium-dose MLWDH-treated group, MLWDH-H: high-dose MLWDH-treated group; Vitamin E: Vitamin E-treated group. Quantitative data are expressed as mean ± SD (n = 8). Statistical comparisons were performed by one-way ANOVA with the following significance thresholds: ns, no significant, * *p* < 0.05, ** *p* < 0.01, *** *p* < 0.001, versus model group.

Comprehensive analyses integrating network pharmacology and molecular docking revealed that MLWDH mediates its effects primarily through regulation of PI3K/AKT signaling, which is a critical regulatory pathway implicated in reproductive protection. To strengthen the mechanistic evidence, PI3K/AKT pathway components were quantitatively assessed at both gene and protein levels to obtain a comprehensive expression profile. CP administration significantly downregulated PI3K and AKT mRNA (14th day: PI3K: *p* < 0.001, η^2^ = 0.56, AKT: *p* < 0.001, η^2^ = 0.49, Figure 7A,B; 28th day: PI3K: *p* < 0.001, η^2^ = 0.72, AKT: *p* < 0.001, η^2^ = 0.76, Figure 8A,B) and protein levels (14th day: p-PI3K/PI3K: *p* < 0.001, η^2^ = 0.80, p-AKT/AKT: *p* < 0.001, η^2^ = 0.62, Figure 7C–G; 28th day: p-PI3K/PI3K: *p* < 0.001, η^2^ = 0.79, p-AKT/AKT: *p* < 0.001, η^2^ = 0.49, Figure 8C–G). Treatment with MLWDH markedly activated the expression of PI3K and AKT. The MLWDH-H group exhibited the most robust upregulation of PI3K/AKT signaling, exceeding the effects observed in the Vitamin E group. Immunofluorescence staining further confirmed increased fluorescence intensity of phosphorylated PI3K (14th day: *p* < 0.001, η^2^ = 0.76; 28th day: *p* < 0.001, η^2^ = 0.75) and phosphorylated AKT (14th day: *p* < 0.001, η^2^ = 0.79; 28th day: *p* < 0.001, η^2^ = 0.65) in MLWDH-treated testes, supporting functional activation of the pathway (Figure 7H–J and Figure 8H–J). These findings reveal that MLWDH activates the PI3K/AKT pathway in OA mice.

### 2.7. MLWDH Enhances Antioxidant Defense via the Nrf2/ARE Signaling

**Figure 9:**
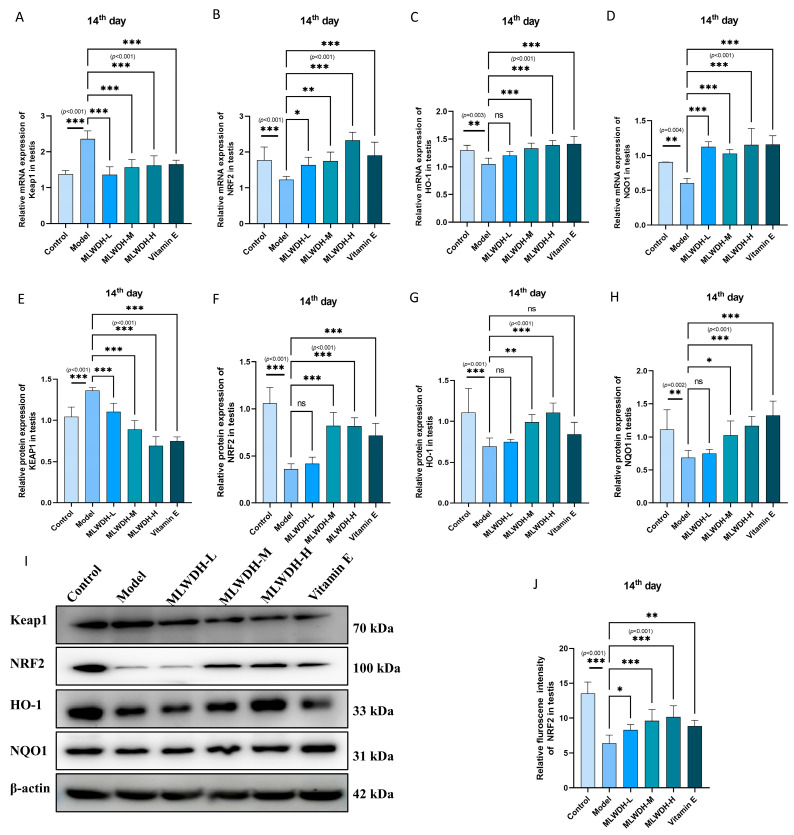

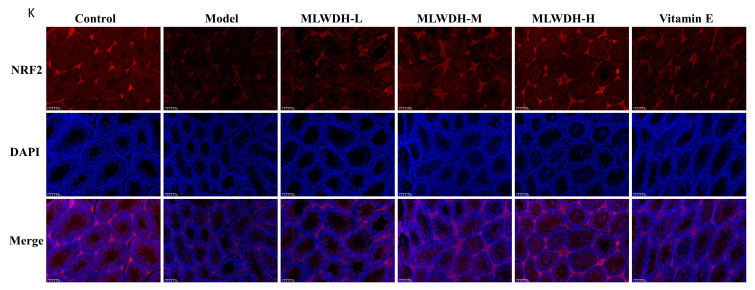
Effects of MLWDH on Nrf2 signaling in OA mice on day 14 after modeling. Keap1, Nrf2, HO-1 and NQO1 mRNA expression in testis (**A**–**D**). Representative immunoblots of Keap1, Nrf2, HO-1 and NQO1 expression (**I**). Quantification of Keap1 (**E**), Nrf2 (**F**), HO-1 (**G**) and NQO1 (**H**) expression, normalized to GAPDH. Immunofluorescent staining of Nrf2 (red) in testis sections (**K**). Quantification of Nrf2 (**J**) protein expression in immunofluorescence, Scale bar = 100 μm. DAPI: blue, target proteins: red, scale bars: white. Control: control group, MLWDH-L: low-dose MLWDH-treated group, MLWDH-M: medium-dose MLWDH-treated group, MLWDH-H: high-dose MLWDH-treated group; Vitamin E: Vitamin E-treated group. Quantitative data are expressed as mean ± SD (n = 8). Statistical comparisons were performed by one-way ANOVA with the following significance thresholds: ns, no significant, * *p* < 0.05, ** *p* < 0.01, *** *p* < 0.001, versus model group.

**Figure 10:**
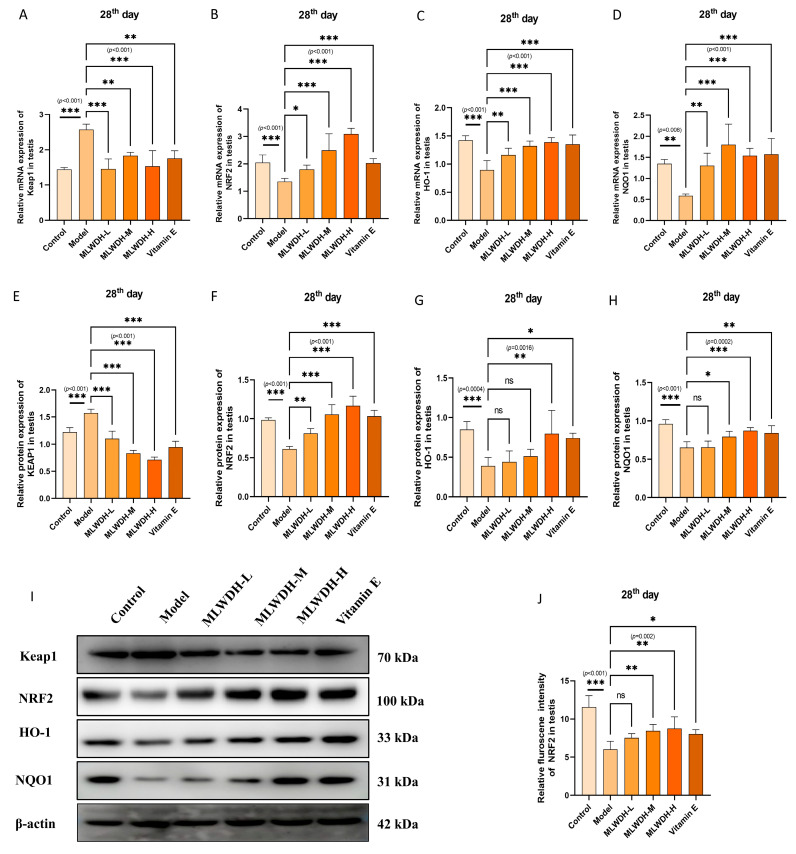

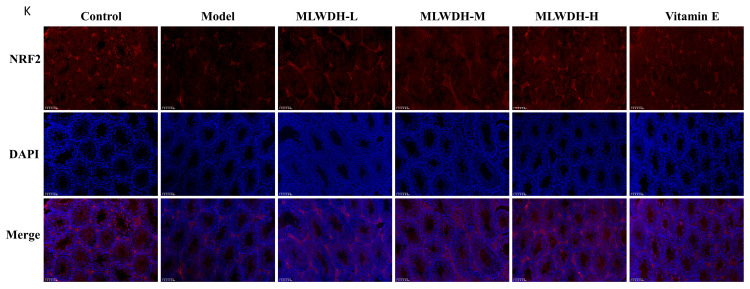
Effects of MLWDH on Nrf2 signaling in OA mice on day 28 after modeling. Keap1, Nrf2, HO-1 and NQO1 mRNA expression in testis (**A**–**D**). Representative immunoblots of Keap1, Nrf2, HO-1 and NQO1 expression (**I**). Quantification of Keap1 (**E**), Nrf2 (**F**), HO-1 (**G**) and NQO1 (**H**) protein expression, normalized to GAPDH. Immunofluorescent staining of Nrf2 (red) in testis sections (**K**), Scale bar = 100 μm. DAPI: blue, target proteins: red, scale bars: white. Quantification of Nrf2 (**J**) expression in immunofluorescence. Control: control group, MLWDH-L: low-dose MLWDH-treated group, MLWDH-M: medium-dose MLWDH-treated group, MLWDH-H: high-dose MLWDH-treated group; Vitamin E: Vitamin E-treated group. Quantitative data are expressed as mean ± SD (n = 8). Statistical comparisons were performed by one-way ANOVA with the following significance thresholds: ns, no significant, * *p* < 0.05, ** *p* < 0.01, *** *p* < 0.001, versus model group.

**Figure 11:**
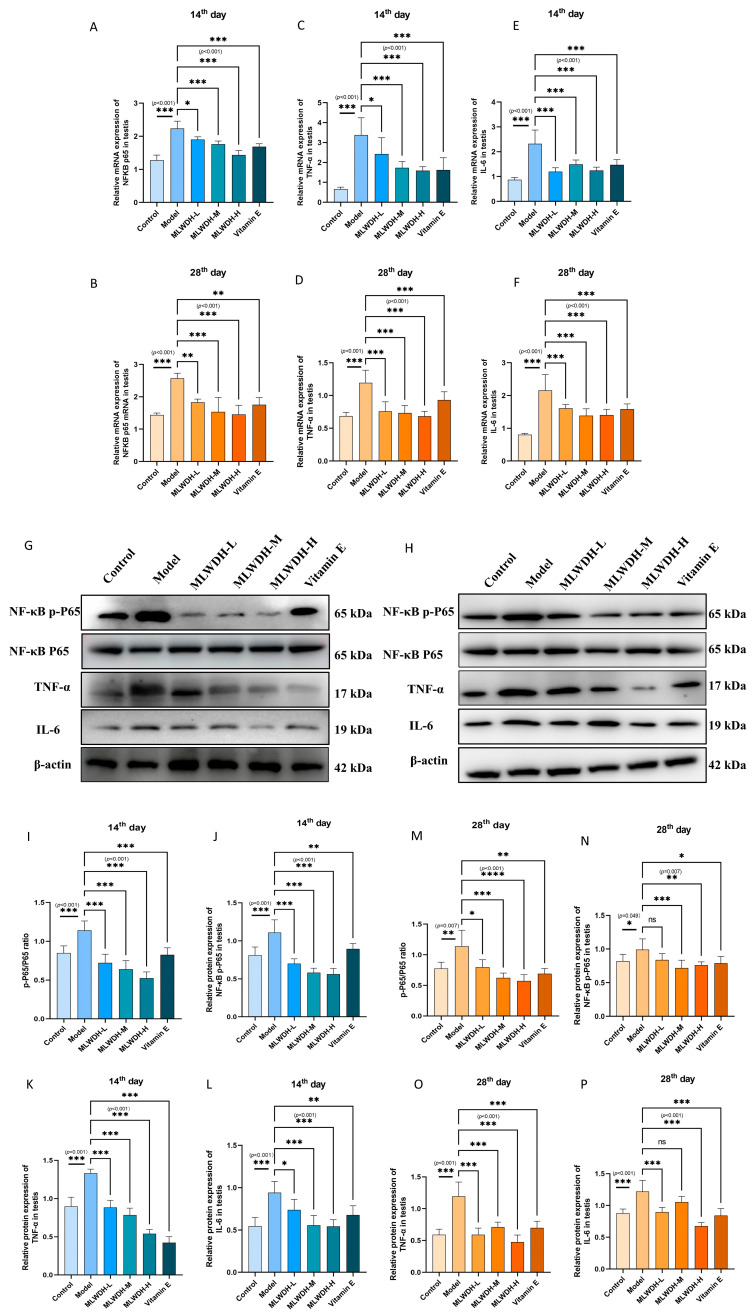
Effects of MLWDH on NF-κB signaling in OA mice on day 14 and 28 after modeling. NF-κB (**A**,**B**), TNF-α (**C**,**D**) and IL-6 (**E**,**F**) mRNA levels in testis. Representative immunoblots of NF-κB, TNF-αand IL-6 expression on day 14 (**G**) and 28 (**H**) after modeling. Quantitative analysis of NF-κB, TNF-αand IL-6 protein expression on day 14 (**I**–**L**) and 28 after modeling (**M**–**P**), normalized to GAPDH. 14th day: on day 14 after modeling; 28th day: on the day 28 after modeling. Quantitative data are expressed as mean ± SD (n = 8). Statistical comparisons were performed by one-way ANOVA with the following significance thresholds: ns, no significant, * *p* < 0.05, ** *p* < 0.01, *** *p* < 0.001, versus model group.

To explore downstream antioxidant mechanisms associated with PI3K/AKT activation, quantitative PCR and immunoblotting were employed to assess the Nrf2/ARE signaling pathway (Figure 9 and Figure 10). CP administration markedly suppressed Nrf2, HO-1, and NQO1 expression while upregulating Keap1, consistent with impaired antioxidative defense (Nrf2 mRNA, *p* < 0.001, η^2^ = 0.62; HO-1 mRNA, *p* = 0.003, η^2^ = 0.63; NQO1 mRNA, *p* = 0.004, η^2^ = 0.60; Keap1 mRNA, *p* < 0.001, η^2^ = 0.77; Figure 9A–D). Similar trends were confirmed at the protein level (Day 14: Nrf2, *p* < 0.001, η^2^ = 0.80; HO-1, *p* < 0.001, η^2^ = 0.58; NQO1, *p* = 0.002, η^2^ = 0.63; Keap1, *p* < 0.001, η^2^ = 0.88; Figure 9E–H). Importantly, Day 28 results followed the same trend, indicating sustained activation of antioxidant signaling (Figure 10). MLWDH administration dose-dependently ameliorated these alterations, evidenced by upregulated expression of Nrf2, HO-1, and NQO1, coupled with reduced Keap1 levels. High-dose MLWDH elicited the most pronounced activation of the antioxidant axis, surpassing Vitamin E. Immunofluorescence analysis further revealed a distinct enhancement in Nrf2 fluorescence intensity in the testicular tissue following MLWDH treatment, consistent with upregulation of antioxidant gene expression (Figure 10F and Figure 11F). These findings indicate that the protective properties of MLWDH may be attributed to its enhancement of intrinsic antioxidant defense mechanisms, potentially mediated via activating the Nrf2/ARE signaling.

### 2.8. MLWDH Mitigates Testicular Inflammation via Modulation of NF-κB Signaling

Considering the intricate connection between redox imbalance and inflammatory pathogenesis in OA, we investigated the NF-κB signaling cascade along with critical pro-inflammatory mediators (IL-6 and TNF-α) to clarify the inflammation-regulating effects of MLWDH (Figure 11). CP exposure resulted in significant increases in NF-κB p65, IL-6, and TNF-α expression at both mRNA (14th day: NF-κB p65, *p* < 0.001, η^2^ = 0.86; IL-6, *p* < 0.001, η^2^ = 0.75; TNF-α, *p* < 0.001, η^2^ = 0.74; 28th day: NF-κB p65, *p* < 0.001, η^2^ = 0.71; IL-6, *p* < 0.001, η^2^ = 0.73; TNF-α, *p* < 0.001, η^2^ = 0.63) and protein levels (14th day: NF-κB p-P65, *p* < 0.001, η^2^ = 0.82; IL-6, *p* < 0.001, η^2^ = 0.67; TNF-α, *p* < 0.001, η^2^ = 0.76; 28th day: NF-κB p-P65, *p* = 0.049, η^2^ = 0.69; IL-6, *p* < 0.001, η^2^ = 0.77; TNF-α, *p* < 0.001, η^2^ = 0.79), consistent with a robust inflammatory response. Importantly, MLWDH treatment, particularly at the high dose, significantly attenuated the CP-induced elevations of NF-κB p65, IL-6, and TNF-α at both the transcriptional and protein levels, indicating its anti-inflammatory activity.

## 3. Discussion

OA, a major cause of male fertility, is characterized by diminished sperm count and motility with elevated morphological defects [51]. Conventional therapies for OA remain limited in efficacy and are often associated with adverse effects, highlighting the need for safer and more effective interventions. TCM offers multitarget pharmacological effects in male reproductive disorders, with LWDH widely applied for treating infertility [21,22,52]. MLWDH, an optimized version of classical LWDH, incorporates additional herbs with complementary pharmacological properties to enhance its antioxidative and reproductive-protective effects. Specifically, *Paeonia lactiflora Pall* (Baishao) has been reported to suppress testicular cell apoptosis, restore reproductive hormone balance, and mitigate reproductive toxicity induced by *tripterygium wilfordii* [53]. *Bupleurum chinense DC* (Chaihu) and *Scolopendra subspinipes mutilans* L. *Koch* (Wugong) exert anti-inflammatory activities and have been investigated for their potential in improving reproductive disorders, including erectile dysfunction [54,55,56]. Bioactive constituents from *Gallus gallus domesticus Brisson* (Jineijin) were shown to attenuate oxidative stress and inflammation by regulating the Nrf2 signaling pathway, which is relevant for testicular protection [57]. Moreover, *Lycium barbarum* L. (Gouqi) has been reported to exert antioxidative and reproductive-protective effects. Its active constituents were shown to regulate MDA, SOD, and GSH levels, thereby suppressing oxidative stress, inhibiting spermatogenic cell apoptosis, and promoting the secretion of FSH and testosterone [58,59,60]. *Cinnamomum cassia* (L.) *J. Presl* (Rougui) has been demonstrated to elevate plasma testosterone, alleviate testicular histopathology, and improve testis index in male rodents [31,61]. *Citrus medica* L. *var. sarcodactylis* (*Noot.*) *Swingle* (Foshou) exhibits strong antioxidant and anti-inflammatory effects, suppressing IL-6, TNF-α, and NF-κB signaling [62,63]. *Angelica sinensis* (*Oliv.*) *Diels* (Danggui) exerts immunomodulatory and anti-inflammatory actions, and purified compounds derived from Danggui can activate ARE-mediated antioxidant gene expression while suppressing pro-inflammatory mediators [58,60,64]. Collectively, these pharmacological insights provide a mechanistic basis for the superior efficacy of MLWDH compared to the classical LWDH, which is further summarized in Table 3.

Although MLWDH has shown promising clinical outcomes, the mechanistic basis of treating OA remains unclear. Here, we demonstrated its protective effects in a CP-induced OA model and explored the underlying mechanisms. MLWDH dose-dependently restored spermatogenic quality, mitigating CP-induced damage. It also restored serum FSH, LH, and testosterone levels, suggesting that MLWDH alleviates CP-induced gonadal axis dysfunction. FSH and LH regulate the function and maturation of Sertoli and Leydig cells, respectively, thereby supporting testosterone production, which is essential for normal spermatogenesis [65,66,67]. The recovery of these hormones is consistent with the observed improvements in sperm quality, suggesting that MLWDH may alleviate CP-induced reproductive injury by preserving the functional integrity of testicular cells. Histological analysis further confirmed that MLWDH preserved seminiferous tubule architecture against CP-induced disruption.

Having established that MLWDH’s efficacy is primarily linked to structural preservation and hormonal regulation, we sought to evaluate its overall therapeutic potential against conventional treatments. Notably, MLWDH demonstrated superior pharmacological effects compared to classical LWDH and vitamin E, enhancing sperm parameters, hormone regulation, and antioxidant defenses.

Excessive accumulation of ROS contributes critically to oxidative damage, which is increasingly recognized as a fundamental mechanism underlying the development of OA [68,69]. ROS impair male reproductive health by disrupting spermatogenesis, reducing sperm concentration and motility, and impairing hormonal regulation [70]. Due to elevated oxygen consumption and significant metabolic activity, the male reproductive system is particularly vulnerable to oxidative insults, which are implicated in 30–80% of male infertility cases [71,72]. Excessive oxidative stress not only damages sperm membrane but also depletes endogenous antioxidant defenses and triggers inflammatory cascades, ultimately leading to germ cell damage [68,71]. Consistent with previous reports, CP administration in the OA model increased oxidative stress biomarkers, notably MDA, alongside a significant decline in endogenous antioxidants such as GSH, NO and SOD [71,73]. MLWDH treatment significantly reversed these alterations by lowering MDA, restoring GSH, SOD and NO, which indicated enhanced endogenous antioxidant capacity and attenuated ROS-mediated damage to sperm membranes. Nrf2 is a primary transcriptional regulator of cellular antioxidant responses, controlling cytoprotective enzyme expression to preserve redox balance. Clinical studies have shown reduced Nrf2 expression in the sperm of oligospermic patients, underscoring its critical role in spermatogenesis and male fertility [6,74]. Upon oxidative challenge, Nrf2 is activated and subsequently dissociates from Keap1, a cytoplasmic repressor of Nrf2. It then translocates into the nucleus, binding to AREs to upregulate key cytoprotective genes (HO-1, NQO1), strengthening cellular defenses [75]. MLWDH intervention notably upregulated Nrf2 at mRNA and protein levels, increased its downstream antioxidant genes HO-1 and NQO1, and suppressed Keap1 in CP-exposed mice. These effects were sustained at both day 14 and day 28 following MLWDH administration, indicating the stability and reliability of MLWDH in activating Nrf2. Collectively, the results suggest that MLWDH protects against oxidative damage by modulation of Nrf2/ARE signaling.

To clarify the material basis of MLWDH against OA, we employed UPLC-HRMS metabolomics. UPLC-HRMS analysis identified 345 constituents, mainly organic acids, flavonoids, and phenolic compounds. Among these, several core compounds such as limonin, esculetin, ferulic acid, oleic acid, and 7-hydroxy-6-methoxy-2H-chromen-2-one have demonstrated bioactivities relevant to oxidative stress, inflammation and spermatogenesis. Limonin activates the Nrf2 pathway, upregulating downstream targets including HO-1 and NQO1, while suppressing NF-κB-mediated inflammation through Sirt1 upregulation [47]. Esculetin exerts antioxidant, anti-inflammatory, and anti-apoptotic effects, protecting against aluminum-induced reproductive toxicity [76]. Ferulic acid alleviates arsenic-induced oxidative stress, testicular injury, and sperm abnormalities via modulation of Nfe2l2, Ppargc1a and StAR expression [77]. Oleic acid functions as an anti-inflammatory immunomodulator, mitigating oxidative stress and inflammation through the Ras/MAPK/PPAR-γ signaling pathway [78]. 7-hydroxy-6-methoxy-2H-chromen-2-one represents a potential anti-inflammatory lead compound [42]. The observed therapeutic effects of MLWDH may arise from both individual compound activities and synergistic interactions among multiple constituents. Network pharmacology analysis predicted PI3K and AKT as core nodes within the compound-target network of MLWDH, with KEGG enrichment analysis indicating the potential involvement of the PI3K/Akt signaling pathways. AKT is a serine-threonine kinase downstream of PI3K with three isoforms. Among them, AKT1 predominates in the testis, where it localizes to spermatogenic and Sertoli cells to maintain germ cell homeostasis and proliferation [79]. Activation of PI3K/AKT signaling enhances sperm motility and concentration while reducing testicular inflammation [3,74,80]. Previous research manifests that stimulation of the PI3K/Akt cascade facilitates Nrf2 nuclear translocation, upregulating antioxidative enzymes and attenuating ROS [81,82]. PI3K/Akt/Nrf2 activation is crucial for redox balance and cellular homeostasis [83]. Conversely, inhibition of PI3K/Akt signaling diminishes Nrf2 activation, reduces expression of antioxidant genes, and compromises cellular defenses, highlighting the central regulatory role of this pathway [84]. Pharmacological studies confirm the PI3K/Akt pathway protects against reproductive damage by upregulating HO-1, SOD and GSH [3,85,86]. Consistent with previous observations, CP exposure significantly suppressed transcriptional (PI3K, AKT) and translational (p-PI3K, p-AKT) components of the PI3K/AKT pathway, as evidenced by RT-qPCR and immunoblotting assays. Notably, treatment with MLWDH effectively reversed this suppression. Immunofluorescence analysis provided further validation, revealing substantially increased immunofluorescent signal intensities of P-PI3K, P-AKT, and Nrf2 on days 14 and 28 post-MLWDH treatment. These findings validate the network predictions and indicate that MLWDH restores PI3K/AKT/Nrf2 signaling, potentially contributing to its effect in oxidative reproductive injury.

Nrf2 and NF-κB p65 are key transcription factors modulating cellular defenses against oxidative stress. Under normal physiological conditions, their activities are balanced to coordinate antioxidative and inflammatory responses [87]. However, excessive ROS production activates NF-κB, thereby initiating pro-inflammatory cascades that further inhibit Nrf2 activity, exacerbating oxidative injury [88,89]. Conversely, sustained NF-κB activation impairs Nrf2-driven transcriptional responses, highlighting their reciprocal interplay [90]. The NF-κB signaling facilitates the transcriptional upregulation of IL-6 and TNF-α, both of which are commonly overexpressed in persistent inflammatory and degenerative pathologies [91]. In this study, CP administration increased NF-κB p65, IL-6, and TNF-α levels in testicular tissues, reflecting an exacerbated inflammation. MLWDH treatment markedly attenuated these increases. This suggests that the formula activates antioxidant pathways via PI3K/AKT/Nrf2 signaling while simultaneously inhibiting the NF-κB axis to exert anti-inflammatory effects. These findings highlight the capacity of MLWDH to restore the Nrf2-NF-κB signaling equilibrium, thereby mitigating both oxidative stress and inflammatory injury in OA pathology.

The present study has several limitations that warrant acknowledgment. First, although network pharmacology and molecular docking identified PI3K/AKT/Nrf2 as a putative mechanism of MLWDH, the absence of direct functional inhibition experiments in vivo means the causal link between MLWDH and PI3K/AKT/Nrf2 activation remains associative. Therefore, direct functional validation using pathway-specific inhibitors (e.g., LY294002 for PI3K [92], MK-2206 for AKT [93]) or genetic approaches (e.g., Nrf2 knockdown) should be incorporated into future in vivo studies to establish causality. Second, our study relies on a cyclophosphamide-induced OA mouse model, which reflects chemically induced testicular injury and oxidative stress. However, human OA is multifactorial, involving genetic, environmental, oxidative, infectious, and chemical factors [94]. Therefore, while MLWDH showed therapeutic efficacy in this chemical model, its effectiveness in OA caused by other etiologies remains uncertain. Future studies using diverse OA models are warranted to validate the translational potential of MLWDH. Third, although MLWDH significantly improved sperm quality and serum reproductive hormones, functional reproductive outcomes (e.g., mating success, litter size, and offspring health) were not assessed. Assessing these endpoints is essential to determine whether improvements in semen parameters translate into meaningful fertility benefits. Accordingly, we plan to incorporate functional reproductive assessments in future studies to strengthen the evidence supporting MLWDH’s therapeutic potential. Finally, as the current work is confined to preclinical data, extrapolation to clinical applications remains premature.

**Table 3:** Comparative composition, molecular targets, and pharmacological actions of MLWDH.

Formula	Herbs	Major Active Compounds	Reported Molecular Targets	Pharmacological Actions
LWDH	Shudihuang	5-Hydroxymethyl-2-furaldehyde, Oleic acid, Succinic acid	PI3K/AKT [95,96], Nrf2, HO-1,NQO1 [97], TNF-α, IL-6 [98]	Anti-inflammatory [95,98], antioxidant [97]
	Shanyurou	Isoleucine, Stearamide, Oleic acid	TNF-α, IL-6 [99]	Anti-inflammatory [99]
	Shanyao	Isoleucine, Tyrosine	PI3K/AKT [96], NF-κB [100]	Anti-inflammatory [96,100]
	Mudanpi	Benzoic acid, Oleic acid	TNF-α, IL-6 [101]	Anti-inflammatory [101]
	Zexie	Stearic acid	PI3K/AKT [102], NF-κB, IL-6 [103]	Anti-inflammatory [102,103]
	Fuling	Oleic acid	TNF-α, NF-κB [104], Nrf2, HO-1 [105]	Immune modulation [104], Anti-inflammatory, Antioxidant [105]
Additional herbs in MLWDH	Baishao	Salicylic acid	PI3K/AKT [106,107,108],Nrf2, NF-κB [109,110]	Anti-inflammatory [106,107], Antioxidant [109,110], Inhibits testicular cell apoptosis, Regulates sex hormone balance [53]
	Chaihu	Oleic acid, 7-hydroxy-6-methoxy-2H-chromen-2-one, Vanillin, Xylitol, Esculetin	PI3K/AKT [111] IL-6,MDA,SOD,GSH [112]	Anti-apoptosis [111], Antioxidant [112,113,114], Erectile dysfunction [56]
	Jineijin	Isoleucine	Nrf2, NQO1, HO-1, MDA, SOD [57]	Antioxidant [57],
	Wugong	Isoleucine, Tyrosine	TNF-α [55]	Anti-inflammatory [55], Erectile dysfunction [54],
	Gouqi	Ferulic acid, Oleic acid, Citric acid, 7-hydroxy-6-methoxy-2H-chromen-2-one	PI3K/AKT [115], Nrf2, Keap1, MDA, SOD, GSH [58], FSH, T, LH, E2 [60]	Promotes cell proliferation [115], Antioxidant [58], Anti-inflammatory [116], Inhibits spermatogenic cell apoptosis [60], Regulates sex hormone balance, Regulates Sertoli cell function, Facilitates spermatogenesis [59]
	Rougui	Oleic acid, Caryophyllene oxide, Methyl cinnamate	Testis index, Testosterone, Estradiol [31], PI3K/AKT, NO, NQO1, Nrf2, MDA [117], IL-6, TNF-α, NO, NF-KB [118]	Ameliorates testicular injury [61], Anti-inflammatory, Antioxidant [117,118], Regulates sex hormone balance [31]
	Foshou	Kojic acid, Oleic acid, 7-hydroxy-6-methoxy-2H-chromen-2-one, Limonin, Isoleucine	IL-6, TNF-α [63], NF-κB [62]	Anti-inflammatory, Antioxidant [63], Immune modulation [62]
	Danggui	Ferulic acid, Azelaic acid, 7-hydroxy-6-methoxy-2H-chromen-2-one, Vanillin, Succinic acid	PI3K/AKT [64], Nrf2 [119], NQO1, TNF-α [120], Keap1 [121], MDA, SOD, NO [30]	Antioxidant [64,119], Improves sperm viability [30], Anti-inflammatory [120]

## 4. Materials and Methods

### 4.1. Preparation of MLWDH

The constituent herbs of MLWDH are presented in Table 4, obtained from Kangmei Pharaceutical (Guangdong, China). Their botanical identities were verified by a qualified expert from the School of Pharmaceutical Sciences, Guangzhou University of Chinese Medicine. The voucher specimen (NO.20231127002) has been archived in the Herbarium of Guangzhou University of Chinese Medicine. The MLWDH was decocted following traditional decoction methods, with reference to previously reported protocols [122,123], ensuring standardization and quality control.

### 4.2. Identification of the Components of MLWDH by UPLC-HRMS

Chemical profiling of MLWDH was performed using an UltiMate 3000 RS ultra-high-performance liquid chromatography integrated with a Q Exactive high-resolution mass spectrometer (UPLC-HRMS, Thermo Fisher Scientific, Shanghai, China) [124]. Samples were prepared by methanol extraction, followed by centrifugation. Chromatographic separation was performed on a Welch AQ-C18 column (2.1 ×100 mm, 1.7 μm, Thermo Fisher Scientific, China) maintained at 35 °C, with a mobile phase consisting of 0.1% formic acid in water (A) and methanol (B). The flow rate was set at 0.3 mL/min, and the injection volume was 5 μL. The gradient program was as follows (time, % A/%B): 0–1.0 min, 98/2; 1.0–5.0 min, 80/20; 5.0–10.0 min, 50/50; 10.0–15.0 min, 20/80; 15.0–27.0 min, 5/95; 28.0–30.0 min, 98/2. Mass spectrometric detection was conducted in both positive and negative modes under data-dependent acquisition across an *m/z* range of 100–1500 Da. Key parameters were: spray voltage 3.5 kV (positive)/−2.8 kV (negative), capillary temperature 300 °C, sheath gas flow 40 arb, auxiliary gas flow 15 arb, S-lens RF level 50. Full MS scans were acquired at a resolution of 70,000 over an *m*/*z* range of 100–1500, followed by data-dependent MS/MS acquisition at a resolution of 17,500. Raw data were processed using Compound Discoverer 3.3 for feature alignment, peak extraction, and compound annotation based on the mzCloud spectral database and relevant literature.

### 4.3. Network Pharmacology Analysis

#### 4.3.1. Collection of MLWDH and OA-Related Targets

Pharmacological compounds of MLWDH were primarily sourced from the Traditional Chinese Medicine Systems Pharmacology Database (TCMSP, https://old.tcmsp-e.com/tcmsp.php, accessed on 26 July 2025) [125], with the exception of *Gallus gallus domesticus Brisson* (Jineijin) and *Scolopendra subspinipes mutilans* L. *Koch* (Wugong), whose bioactive constituents were obtained from the HERB database (http://herb.ac.cn/, accessed on 2 May 2025) [126], due to their absence in the TCMSP database. To ensure both authenticity and pharmacological relevance, a two-step filtering strategy was employed. First, only compounds simultaneously detected by UPLC-HRMS and recorded in TCMSP or HERB for the 14 constituent herbs were retained, thereby minimizing potential analytical artifacts. Second, pharmacokinetic criteria (oral bioavailability ≥ 30% and drug-likeness ≥ 0.18) were applied; compounds not meeting both thresholds were further evaluated by systematic validation through manual screening of studies retrieved from PubMed. Venny 2.1 website (https://bioinfogp.cnb.csic.es/tools/venny/index.html, accessed on 26 July 2025) was employed to intersect the UPLC-HRMS detected compounds with the database-curated dataset, yielding representative bioactive components. The potential targets of the identified consensus components were inferred using the SwissTargetPrediction platform (http://swisstargetprediction.ch/, accessed on 4 May 2025) [127] with a probability threshold ≥ 0. After removing duplicates, a non-redundant target dataset for MLWDH was constructed.

To systematically identify potential therapeutic targets for OA, the GeneCards database (https://www.genecards.org, accessed on 5 May 2025) [128] was searched. As direct retrieval using the keyword “oligoasthenozoospermia” yielded a limited number of targets, additional searches were therefore performed using the related terms “oligozoospermia” and “asthenozoospermia”, given that OA is a clinical condition characterized by the coexistence of these two conditions. The intersection of targets from these two conditions was taken to represent the core pathological basis of OA. In addition, the keyword “spermatogenic dysfunction” was included to capture genes involved in broader spermatogenic abnormalities. After removing duplicates, all identified genes were combined to generate a unified target set, defined as OA-related candidate targets, for subsequent network construction and mechanistic analysis.

#### 4.3.2. Creating a PPI Network and Identifying Core Genes

Protein–protein interaction (PPI) relationships among OA-related candidate targets were identified using the STRING platform (https://string-db.org/, accessed on 7 May 2025) [129]. The analysis was restricted to Homo sapiens with an interaction confidence score threshold of 0.4 (moderate reliability). Interaction data were formatted as TSV and visualized using Cytoscape (v3.10.3). The CytoNCA plugin was utilized to compute centrality metrics, including degree, betweenness, and closeness. Genes with values exceeding the median for degree, betweenness, and closeness were identified as hub nodes. Among these, degree centrality was further used to emphasize the crucial targets.

#### 4.3.3. Constructing Drug-Component-Target Networks

The Drug-Component-Target interaction networks were generated via Cytoscape version 3.10.3 to visualize the hierarchical connections among MLWDH, their constituent compounds, and predicted targets, thereby facilitating the identification of key bioactive ingredients within the network.

#### 4.3.4. Enrichment Analysis

Common targets between MLWDH and OA were identified and subsequently analyzed for Kyoto Encyclopedia of Genes and Genomes (KEGG) pathway enrichment analyses using the Metascape platform (https://metascape.org, accessed on 8 May 2025) [130].

#### 4.3.5. Molecular Docking

Crucial bioactive constituents were identified by analyzing degree values with the cytoNCA plugin. The five leading candidate constituents were prioritized for molecular docking. The 3D structures of PI3K (PIK3CA, PDB ID: 9ASF) and AKT (AKT1, PDB ID: 8UW7) were acquired from the RCSB Protein Data Bank (https://www.rcsb.org/, accessed on 9 May 2025) [131], and the corresponding 2D molecular structures of the selected phytochemicals were retrieved from the PubChem repository (https://pubchem.ncbi.nlm.nih.gov/, accessed on 9 May 2025) [132]. Molecular docking was performed via the CB-Dock2 server, and binding affinities were assessed according to AutoDock Vina scores (https://cadd.labshare.cn/cb-dock2/php/index.php, accessed on 10 May 2025) [133]. Binding energies ≤ −5.0 kcal/mol were adopted to validate a potentially favorable ligand-target interaction. The spatial conformations and binding interfaces between the ligands and receptor proteins were visualized and interpreted by PyMOL (https://www.pymol.org/, accessed on 10 May 2025).

### 4.4. Reagents

Haematoxylin and eosin (H&E) stain was procured from Youtian Biotechnology (Guangzhou, China). The SYBR Green Pro Taq HS Premix (AG11701) was supplied by Accurate Biology (Changsha, China), and RevertAid Reverse Transcriptase (EP0442) was obtained from Thermo Fisher Scientific (NewYork, NY, USA). The Trizol reagent for RNA extraction (9109) was purchased from TAKARA (Shiga, Japan). Enzyme-linked immunosorbent assay (ELISA) kits for testosterone (T), follicle-stimulating hormone (FSH), and luteinizing hormone (LH) quantification in mouse serum were purchased from LabRe (Wuhan, China). Detection kits for Superoxide Dismutase (SOD), Reduced Glutathione (GSH), Nitric Oxide (NO), Malondialdehyde (MDA), and Quick Sperm Stain Kit were sourced from Nanjing Jiancheng Bioengineering Institute (Nanjing, China). Additionally, the protein quantification kit (BL521A) and protein extraction kit (BL401A) were obtained from Biosharp (Beijing, China). Vitamin E was obtained from Zhejiang Medicine (Shaoxing, China).

### 4.5. Animals and Treatment

Specific pathogen-free Kunming mices (male, 20–25 g, 6–8 weeks old) were obtained from the Experimental Animal Center of Guangzhou University of Chinese Medicine (Approval No. SCXK (Yue) 2023-0347; SYXK (Yue) 2023-0068). All animals were kept under a standardized housing environment with an ambient temperature of 22 to 24 °C, relative humidity between 50% and 60%, and a regulated light/dark schedule (12 h/12 h). Ethical approval for all animal procedures was granted by the Ethics Committee for Animal Experiment Center, Guangzhou University of Chinese Medicine (No. 20240508006) approved on May 9, 2024. To induce a model of OA, CP was intraperitoneally injected at 50 mg/kg/day for 7 days [134], using sterile saline as the vehicle, except for the control group. 112 male mice were equally randomized into seven groups (n = 16): control group; model group; MLWDH-H group (20 g/kg); MLWDH-M group (10 g/kg); MLWDH-L group (5 g/kg); LWDH group (20 g/kg); and positive group (Vitamin E, 5 mg/kg) [75]. After seven consecutive days of modeling, mice in the treatment groups received the designated compounds by oral gavage, whereas animals in the control and model groups were administered an equivalent volume of sterile saline daily.

### 4.6. Pathologic Sampling of Mice

On days 14 and 28 following the completion of modeling, eight mice per group were chosen at random and weighed after a 12 h fast. Under anesthesia, blood was collected via orbital extraction immediately prior to euthanasia. The epididymis and testis of the mice were carefully excised and separated after euthanasia. Subsequently, serum samples were harvested and subjected to biochemical analyses for the quantification of T, FSH, LH, MDA, GSH, NO, and SOD. The testicular and epididymal tissues were rinsed with precooled saline to ensure the removal of residual impurities, blotted to remove excess fluid, and subsequently weighed with precision. Of the two testes obtained from each mouse, one was maintained for H&E staining. Another testis was longitudinally divided into two parts: one half was prepared for real-time quantitative PCR (RT-qPCR), while the remaining half was processed for protein isolation and Western blot analysis. Simultaneously, the epididymis was processed to evaluate sperm characteristics, including sperm count, motility, and morphology [135].

### 4.7. Determination of Organ Index

Testicular and epididymal tissues were meticulously dissected and individually weighed. Organ index was derived by normalizing the organ weight to the body weight. Specifically, the testicular organ index was computed using the formula: testicular index (%) = [combined weight of both testes (g)/body weight (g)] × 100%. Similarly, the epididymal index was calculated as: epididymal index (%) = [combined weight of both epididymides (g)/body weight (g)] × 100%.

### 4.8. Evaluation of Sperm Characteristics

The dissected epididymal tissue was minced in 2 mL of sterile physiological saline and maintained at 37 °C for 10 min to facilitate sperm dispersion. The resulting suspension was subsequently used to evaluate sperm count and motility using a hemocytometer, following protocols outlined in previous studies [135]. For the assessment of sperm morphology, a 10 μL aliquot of the prepared sperm suspension was uniformly spread on adhesive glass slides and air-dried at room temperature for morphological analysis. Dried slides were stained with the Quick Sperm Stain Kit, following the standard protocol. Each epididymis underwent triplicate analyses to ensure reproducibility. Two hundred sperm per sample were evaluated microscopically. The proportion of abnormal spermatozoa was calculated based on established morphological criteria. All microscopic evaluations were performed using a Nikon microscope (Nikon, Japan).

### 4.9. Histopathology Analysis

To evaluate testicular histopathological alterations in OA mice, testicular tissues were immersed in 4% paraformaldehyde and preserved at room temperature for 48 h. The testicular tissues were dehydrated, infiltrated with paraffin, and then sectioned into slices (5 μm) using a microtome. Subsequently, testicular sections were dewaxed, rehydrated, and subjected to H&E staining for 10 min. After dehydration, the sections were sealed with neutral resin. Histopathological analysis was conducted using a light microscope (400×), while images were captured with an integrated digital camera affixed to the microscope (Nikon, Japan).

### 4.10. Serum Biomarkers Analysis

Blood samples were collected through ocular puncture in the mice and centrifuged to separate the serum (6000 rpm, 15 min). The serum levels of testosterone, FSH, and LH were quantified via ELISA. Additionally, levels of MDA, GSH, SOD, and NO were assessed by biochemical assay kits under standardized protocols.

### 4.11. RT-qPCR Analysis

Fresh testicular tissue sample (20–30 mg) was thoroughly homogenized in Trizol reagent, promptly preserved at −80 °C. Chloroform was added to the Trizol homogenate for phase separation, followed by RNA precipitation with isopropanol and ethanol. Total RNA was subsequently converted to complementary DNA using oligo dT18 primers and RevertAid Reverse Transcriptase (Thermo Fisher Scientific, USA). Quantification of mRNA expression for PI3K, AKT, Nrf2, Keap1, HO-1, NQO1, SOD, NO, TNF-α, and IL-6 was performed via RT-qPCR. Glyceraldehyde-3-phosphate dehydrogenase (GAPDH) was employed as the endogenous control for gene expression normalization. Relative gene expression was quantified through RT-qPCR with a standard curve-based approach. All primer sequences were generated with ABI Primer Express software and listed in Table 5.

### 4.12. Western Blot Analysis

Proteins were isolated from testicular tissues using a commercial extraction kit (Beyotime, Shanghai, China), and their concentrations were determined by the BCA assay method (Beyotime, Shanghai, China) following the manufacturer’s protocol. Protein samples (30 μg) were separated by 10% SDS-PAGE and transferred onto polyvinylidene difluoride (PVDF) membranes. After blocking with 5% bovine serum albumin (BSA), the membranes were incubated overnight at 4 °C with specific primary antibodies. The next day, membranes were rinsed thrice and exposed to secondary antibodies (HRP-labeled anti-rabbit IgG). Immunoreactive images were detected using the ECL Plus chemiluminescence detection system (Biosharp, Hefei, China). Antibodies (PI3K, Phospho-PI3K, AKT, Phospho-AKT, HO-1, TNF-α) and secondary antibodies (HRP-conjugated anti-rabbit IgG) were sourced from Abmart (Shanghai, China). Other antibodies (Nrf2, KEAP1, NQO1, IL-6, NF-κB p65, Phospho-NF-κB p65, and β-actin) were obtained from Affinity Biosciences (Changzhou, China).

### 4.13. Immunofluorescence Stain

Testicular sections embedded in paraffin were initially treated with xylene to remove the embedding medium, followed by sequential dehydration in descending ethanol concentrations (100%, 95%, and 75%). To minimize nonspecific antibody interactions, tissue slides were pretreated with goat serum (10%) at 37 °C for 1 h. Thereafter, they were exposed to primary antibodies in a moist chamber for a whole night. On the following day, secondary antibodies labeled with fluorophores were maintained at 37 °C for 1 h. For staining p-PI3K, p-AKT, and Nrf2, tissue sections were sequentially incubated with corresponding primary antibodies, followed by labeling with distinct fluorophore-conjugated secondary antibodies. Secondary antibodies (Goat Anti-Rabbit IgG (H+L) FITC-conjugated, Goat Anti-Rabbit IgG (H+L) Fluor594-conjugated) were obtained from Affinity Biosciences (Changzhou, China). Fluor594-conjugated anti-rabbit IgG was labeled for p-AKT and Nrf2, and FITC-conjugated goat anti-rabbit IgG was labeled for p-PI3K. Nuclear counterstaining was performed with DAPI. Fluorescence images were acquired using a microscope (Nikon Corporation, Tokyo, Japan), and signal intensities were quantified with ImageJ software (version 1.52).

### 4.14. Statistical Analysis

To ensure reproducibility, all experiments were conducted independently at least three times, with data from a representative replicate are presented. Statistical data were analyzed by GraphPad Software (version 9.0). Quantitative results are expressed as mean ± standard deviation (SD). Statistical significance was defined as *p* < 0.05. In addition to *p*-values, effect sizes were calculated as eta squared (η^2^) based on ANOVA outputs, using the formula η^2^ = SS (effect)/SS (total) [136]. According to conventional benchmarks, η^2^ values of 0.01, 0.06, and 0.14 represent small, medium, and large effects, respectively.

## 5. Conclusions

This study highlights MLWDH as a promising multi-component formulation with potential therapeutic relevance for OA. By integrating network pharmacology and in vivo pharmacological evaluation, we demonstrated that MLWDH ameliorates sperm quality and testicular injury by modulating the PI3K/AKT/Nrf2 antioxidant axis and suppressing NF-κB-driven inflammatory responses. Notably, the optimized herbal composition of MLWDH exhibited superior efficacy compared to the LWDH, underscoring the advantage of rational decoction improvement. Collectively, these findings provide mechanistic insights and pharmacological evidence supporting the potential of MLWDH for further investigation in male reproductive disorders.

## Figures and Tables

**Table 1:** Information on representative MLWDH ingredients identified by both UPLC-HRMS analysis and database screening.

NO.	Molecular ID	Molecular Name	Formula	Ion	RT [min]
1	MOL000068	Isoleucine	C_6_H_13_NO_2_	[M+H]^+1^	2.45
2	MOL000360	Ferulic acid	C_10_H_10_O_4_	[M+H-H_2_O]^+1^	11.82
3	MOL000103	Benzoic acid	C_7_H_6_O_2_	[M-H]^−1^	9.65
4	MOL000056	Tyrosine	C_9_H_11_NO_3_	[M+H]^+1^	3.00
5	MOL000748	5-Hydroxymethyl-2-furaldehyde	C_6_H_6_O_3_	[M+H]^+1^	5.96
6	MOL005131	Kojic acid	C_6_H_6_O_4_	[M+H]^+1^	7.30
7	MOL003940	Stearamide	C_18_ H_37_NO	[M+H]^+1^	22.62
8	MOL001314	Azelaic acid	C_9_H_16_O_4_	[M-H]^−1^	13.33
9	MOL000675	Oleic acid	C_18_H_34_O_2_	[M-H]^−1^	22.72
10	MOL001801	Salicylic acid	C_7_H_6_O_3_	[M-H]^−1^	12.87
11	MOL001456	Citric acid	C_6_H_8_O_7_	[M-H]^−1^	2.46
12	MOL000040	7-hydroxy-6-methoxy-2H-chromen-2-one	C_10_H_8_O_4_	[M+H]^+1^	11.47
13	MOL002003	Caryophyllene oxide	C_15_H_24_O	[M+H]^+1^	15.68
14	MOL000635	Vanillin	C_8_H_8_O_3_	[M+H]^+1^	10.51
15	MOL004712	Xylitol	C_5_H_12_O_5_	[M+H-H_2_O]^+1^	1.36
16	MOL000249	Methyl cinnamate	C_10_H_10_O_2_	[M+H+MeOH]^+1^	10.26
17	MOL004666	Ethyl protocatechuate	C_9_H_10_O_4_	[M+H]^+1^	11.86
18	MOL003959	Limonin	C_26_H_30_O_8_	[M+H]^+1^	14.22
19	MOL003837	Esculetin	C_9_H_6_O_4_	[M-H]^−1^	9.90
20	MOL000346	Succinic acid	C_4_ H_6_O_4_	[M-H]^−1^	3.15

**Table 2:** Bing energy table of key components and core proteins.

	Ferulic Acid	7-Hydroxy-6-methoxy-2H-chromen-2-one	Limonin	Esculetin	Oleic Acid
PIK3CA	−6.7 kcal/mol	−6.5 kcal/mol	−10.4 kcal/mol	−7 kcal/mol	−6.3 kcal/mol
AKT1	−6.4 kcal/mol	−6.9 kcal/mol	−9.2 kcal/mol	−6.9 kcal/mol	−5.8 kcal/mol

**Table 4:** Contents of MLWDH.

Chinese Name	Accepted Name	Weight (g)	Medicinal Part
Shudihuang	*Rehmannia glutinosa (Gaertn.) DC.*	30	Prepared root
Shanyurou	*Cornus officinalis Siebold & Zucc.*	10	Flesh of the fruit (dried)
Shanyao	*Dioscorea polystachya Turcz*	30	Tuber (dried)
Mudanpi	*Paeonia suffruticosa Andrews*	10	Root bark (dried)
Zexie	*Alisma orientale (Sam.) Juzep*	10	Tuber (dried)
Fuling	*Poria cocos (Schw.) Wolf*	15	Sclerotium (fungal body, dried)
Baishao	*Paeonia lactiflora Pall*	30	Root
Chaihu	*Bupleurum chinense DC*	10	Root
Jineijin	*Gallus gallus domesticus Brisson*	20	Inner lining of the gizzard (dried)
Wugong	*Scolopendra subspinipes mutilans* L. *Koch*	3	Entire dried body
Gouqi	*Lycium barbarum L*	10	Fruit (dried)
Rougui	*Cinnamomum cassia* (L.) *J. Presl*	5	Bark
Foshou	*Citrus medica* L. *var. sarcodactylis (Noot.) Swingle*	10	Fruit (dried or fresh)
Danggui	*Angelica sinensis (Oliv.) Diels*	10	Root (dried)

**Table 5:** Primers for RT-qPCR.

Gene	Forward	Reverse
PI3K	5′-GAAGAAGCTGAACGAGTCGC-3′	5′-CCCGACATTCCACGTCTTCT-3′
AKT1	5′-CTGCTCCTAGTCCACCACCT-3′	5′-AGAGACCTCCATTATCGCTACC-3′
Nrf2	5′-AGACATTCCCATTTGTAGATGACC-3′	5′-CTCCAGAcGAGCTATTGAGGGACT-3′
KEAP1	5′-TCGAAGGCATCCACCCTAAG-3′	5′-CTCGAACCACGCTGTCAATCT-3′
HO-1	5′-ACAGAGGAACACAAAGACCAGAGT-3′	5′-GTGTCTGGGATGAGCTAGTGC-3′
NQO1	5′-TGGCCGAACACAAGAAGCTG-3′	5′-GCTACGAGCACTCTCTCAAACC-3′
NF-κB p65	5′-GTTCACAGACCTGGCATCTG-3′	5′-CCTGTCACCAGGCGAGTTAT-3′
TNF-α	5′-TGTCTCAGCCTCTTCTCATTCC-3′	5′-GGTCTGGGCCATAGAACTGAT-3′
IL-6	5′-CCAAGAGGTGAGTGCTTCCC-3′	5′-CTGTTGTTCAGACTCTCTCCCT-3′
GAPDH	5′-GTGGACCTCATGGCCTACAT-3′	5′-TGTGAGGGAGATGCTCAGTG-3′

## Data Availability

The original contributions presented in this study are included in the article/supplementary material. Further inquiries can be directed to the corresponding author.

## Supplementary Materials

The following supporting information can be downloaded at https://www.mdpi.com/article/10.3390/ph18091363/s1.

## Abbreviations

The following abbreviations are used in this manuscript:

ARE	Antioxidant Response Elements
CP	Cyclophosphamide
FSH	Follicle-stimulating Hormone
GSH	Glutathione
HO-1	Heme Oxygenase-1
UPLC-HRMS	Ultra performance liquid chromatography-high resolution mass spectrometry
Keap1	Kelch-like ECH-associated Protein 1
KEGG	Kyoto encyclopedia of gene and genomes
LH	Luteinizing Hormone
LWDH	Liuwei Dihuang Decoction
MDA	Malondialdehyde
MLWDH	Modified Liuwei Dihuang Decotion
NO	Nitric Oxide
NOS	Nitric Oxide Synthase
Nrf2	Nuclear Factor Erythroid 2-Related Factor 2
NS	Normal Saline
OA	Oligoasthenozoospermia
PI3K	Phosphatidylinositol 3-kinase
P-PI3K	Phosphorylated PI3K
PPI	protein–protein interaction
ROS	Reactive Oxygen Species
SCs	Sertoli Cells
SOD	Superoxide Dismutase
TCM	Traditional Chinese Medicine
TCMSP	Traditional Chinese Medicine Systems Pharmacology Database
