# Supplementary material for: Modified Liuwei Dihuang Decoction Ameliorates Oligoasthenozoospermia in Mice via Modulation of the PI3K/AKT/Nrf2 Signaling Pathway
Source: Pharmaceuticals (Basel). 2025 Sep 12;18(9):1363. doi: 10.3390/ph18091363 (PMC12472625; doi:10.3390/ph18091363)
Supplement: Supplementary file 1 [file pharmaceuticals-18-01363-s001.zip › pharmaceuticals-3812787-supplementary.pdf]

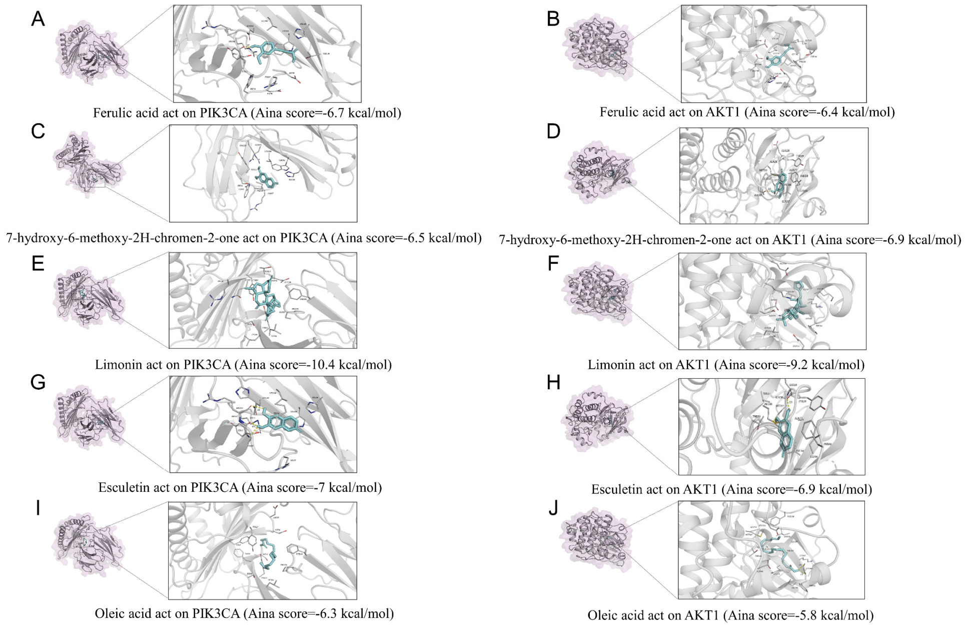

Figure S1. Molecular docking analysis of MLWDH bioactive compounds with PI3K/AKT signaling. The 3D schematic diagram of ferulic acid with PIK3CA(A) and AKT1(B); The 3D schematic diagram of 7-hydroxy-6-methoxy-2H-chromen-2-one with PIK3CA(C) and AKT1(D); The 3D schematic diagram of limonin with PIK3CA (E) and AKT1(F), The 3D schematic diagram of esculetin with PIK3CA (G) and AKT1(H), The 3D schematic diagram of oleic acid with PIK3CA(I) and AKT1(J)
